# Correlation of MRI Visual Scales with Neuropsychological Profile in Mild Cognitive Impairment of Parkinson's Disease

**DOI:** 10.1155/2017/7380102

**Published:** 2017-03-19

**Authors:** Luiz Felipe Vasconcellos, João Santos Pereira, Marcelo Adachi, Denise Greca, Manuela Cruz, Ana Lara Malak, Helenice Charchat-Fichman, Mariana Spitz

**Affiliations:** ^1^Movement Disorders Unit, Neurology Service, Pedro Ernesto University Hospital, State University of Rio de Janeiro, Rio de Janeiro, RJ, Brazil; ^2^Radiology Department, Hospital Central da Polícia Militar, Rio de Janeiro, RJ, Brazil; ^3^Psychology Department, Pontifical Catholic University of Rio de Janeiro (PUC-Rio), Rio de Janeiro, RJ, Brazil

## Abstract

Few studies have evaluated magnetic resonance imaging (MRI) visual scales in Parkinson's disease-Mild Cognitive Impairment (PD-MCI). We selected 79 PD patients and 92 controls (CO) to perform neurologic and neuropsychological evaluation. Brain MRI was performed to evaluate the following scales: Global Cortical Atrophy (GCA), Fazekas, and medial temporal atrophy (MTA). The analysis revealed that both PD groups (amnestic and nonamnestic) showed worse performance on several tests when compared to CO. Memory, executive function, and attention impairment were more severe in amnestic PD-MCI group. Overall analysis of frequency of MRI visual scales by MCI subtype did not reveal any statistically significant result. Statistically significant inverse correlation was observed between GCA scale and Mini-Mental Status Examination (MMSE), Montreal Cognitive Assessment (MoCA), semantic verbal fluency, Stroop test, figure memory test, trail making test (TMT) B, and Rey Auditory Verbal Learning Test (RAVLT). The MTA scale correlated with Stroop test and Fazekas scale with figure memory test, digit span, and Stroop test according to the subgroup evaluated. Visual scales by MRI in MCI should be evaluated by cognitive domain and might be more useful in more severely impaired MCI or dementia patients.

## 1. Introduction

Parkinson's disease (PD) is characterized by motor and nonmotor features. Among nonmotor symptoms, cognitive impairment results in significant morbidity and mortality; therefore early diagnosis is essential for appropriate treatment and subsequent reduction of the disease burden [1]. Some variables related to increased risk of cognitive decline have been suggested, such as rigid-akinetic phenotype, aging, female gender, anticholinergic use, and mild cognitive impairment [2]. Neuropsychological risk factors to PD dementia (PDD) are impaired, semantic fluency and visuospatial and executive function [2, 3].

PD-Mild Cognitive Impairment (PD-MCI) represents a cognitive impairment not severe as to interfere in daily activities and does not fulfill criteria for dementia in a patient with clinically established PD. MCI can be classified, according to the main area of impairment, as amnestic (memory primarily affected) or nonamnestic (other cognitive function). These differences are beyond the main cognitive domain as well prognosis (higher risk for conversion to dementia) [3, 4].

Movement Disorders Society (MDS) criteria for PD-MCI and PDD seem to be the most suitable to diagnose such conditions. These criteria can be rated as level 1 or 2 depending on cognitive assessment. Level 2 (which has a higher specificity) requires neuropsychological testing of multiple domains [4, 5].

PD patients have increased risk for developing cognitive impairment and it is recommended to periodically perform a neuropsychological assessment in these patients. It is important to emphasize that executive function could worsen in OFF phase, so use of levodopa is mandatory preceding neuropsychological assessment [6].

Structural abnormalities revealed by brain magnetic resonance imaging (MRI) may be related to cognitive impairment in PD-MCI or PDD. Besides MRI, other neuroimaging tools used for this purpose include PET or SPECT, available only in specialized centers at a high cost. Conventional MRI is widely available, cost-effective, and easy to perform despite limited sensitivity and specificity. MRI visual rating scales which are useful in cognitive impairment include global cortical atrophy (GCA), medial temporal atrophy (MTA), and Fazekas scale [7]. Atrophy of several brain structures has been associated with cognitive impairment in PD, as memory with MTA and dysexecutive syndrome with cortical-subcortical structures. Comparing PDD with Alzheimer's disease (AD), abnormalities of MTA can be detected in both conditions, but with more expressive reduction in AD. A lesser degree of MTA in PD-MCI has also reported, even in cognitively intact PD patients. Possibly the association of PD-MCI and MTA represents a higher risk to develop PDD [8, 9]. One important factor to consider when analyzing previous MRI studies correlating PD-MCI with visual scales is how the diagnosis of MCI was established, as more specific criteria for PD-MCI have been recently defined. Therefore, a patient formerly diagnosed with MCI could in fact have PDD.

Studies with GCA, MTA, and white matter hyperintensity (WMH) evaluated by the Fazekas scale in PD-MCI are rare and present doubtful results.

We evaluated in the present study if these structural changes detected by MRI could be related to MCI subtype and its frequency in PD patients comparing with control group (CO).

## 2. Methods

PD patients were sequentially and prospectively recruited from 3 outpatient Movement Disorders Clinics during 18 months. All fulfilled UK Brain Bank Criteria for PD [10]. Control group (CO) consisted of individuals without any neurologic or psychiatric diagnoses being family members of patients or hospital employees. All patients and controls were between 50 and 75 years old and had more than 4 years of education. Both groups consisted of individuals with MCI according to neuropsychology evaluation. They underwent blood screening exams, including thyroid hormones, B12 vitamin, folic acid, and venereal disease research test and brain MRI. They were excluded in the case of any abnormality in the exams, for example, any kind of stroke in the MRI. The use of benzodiazepine and anticholinergic was another exclusion criterion. Subjects previously diagnosed with dementia and depression were also excluded.

The assessment consisted of neurologic examination with several clinical scales:

- Motor assessment: Hoehn-Yahr Scale and Unified Parkinson's Disease Rating Scale (UPDRS).
- Quality of life: PDQ-39.
- Activities of daily living: Schwab-England.
- Total medication: levodopa equivalent dose (LED).
- Criteria for depression and anxiety: DSM IV.

PD patients were classified according to the predominant motor sign: tremulous, akinetic-rigid, or mixed.

Neuropsychology evaluation with the following tests was performed:

- Mini-Mental Status Examination (MMSE).
- Montreal Cognitive Assessment (MoCA).
- Clock Drawing Test.
- Verbal fluency: semantic (animals) and phonemic (letters F, A, and S).
- Stroop test.
- Figure memory test (FMT).
- Trail making test (TMT).
- Digit span (WAIS-III).
- Rey Auditory Verbal Learning Test (RAVLT).
- Hooper Visual Organization Test.
- Beck Depression Inventory (BDI).

PD-MCI was classified according to MDS criteria (Level 2) and CO-MCI according to Forlenza et al., both with −1.5 standard deviation (SD) below the mean [4, 11]. Memory impairment characterized amnestic MCI and impairment of one or more non-memory cognitive domains, including executive function, attention, language and visuospatial skills, characterized nonamnestic MCI.

The medium time lapse between neuropsychological evaluation and MRI was 2 months. MRI scans were performed on a Siemens 1.5-Tesla device. Three-dimensional T1-weighted images were acquired in sagittal orientation employing a 3D-SPGR sequence [TR = 8.8 ms, TE = 4 ms, and matrix = 240 × 240]; 170 slices were collected with 1.0 mm thickness without gap. Axial* FLAIR* images with 5.0 mm thickness [TR = 11.000 ms, TE = 140 ms, and matrix = 512 × 512] were obtained for WM analysis. The radiologist was blinded to the sex, age, and diagnosis of the subjects. The scales evaluated were as follows.

(i) Global Cortical Atrophy (GCA) or Pasquier scale: scored on FLAIR sequence [12].

Score:

  (0) no cortical atrophy;
  (1) mild atrophy (opening of sulci);
  (2) moderate atrophy (volume loss of gyri);
  (3) severe (end-stage) atrophy (“knife blade” atrophy).

(ii) Medial Temporal Lobe Atrophy (MTA) or Scheltens scale: rated on coronal T1-weighted slice through the corpus of the hippocampus, at the level of the anterior pons [13]. In the case of asymmetry, the highest score is the one to consider.

Score:

  (0) no atrophy;
  (1) only widening of choroid fissure;
  (2) also widening of temporal horn of lateral ventricle;
  (3) moderate loss of hippocampal volume (decrease in height);
  (4) severe volume loss of hippocampus;
  <75 years old: score (2) or more is abnormal;
  >75 years old: score (3) or more is abnormal.

(iii) Fazekas scale for WMH lesions: scored on axial FLAIR or T2-weighted images [14].

Score:

  (0) none or a single punctate WMH lesion;
  (1) multiple punctate lesions;
  (2) beginning confluency of lesions (bridging);
  (3) large confluent lesions;
  <70 years old: score (2) or more is considered abnormal;
  >70 years old: score (3) is abnormal.

This study was approved by the ethical committee of all institutions involved, and written informed consent was obtained from all subjects.

### 2.1. Statistical Analysis

Mann–Whitney test was used to compare groups in terms of disease duration, L-dopa equivalent dose, UPDRS-III, PDQ-39 and Schwab-England scores, and Kruskal-Wallis test for demographics characteristics.

The neuropsychological performance of patients and controls was compared by using Kruskal-Wallis test. Spearman correlation was conducted between MRI analysis and neuropsychological tests scores.

Statistical significance was set at *p* < 0.05 for all analyses.

## 3. Results

Seventy-nine PD patients and 92 controls (CO) with MCI were evaluated as potential candidates. Thirty-five PD patients and 48 CO had one or more findings that could be responsible for another cause of cognitive impairment (exclusion criteria). Both groups (44 PD and 44 CO) were classified by neuropsychological profile as MCI amnestic (*n* = 23) or nonamnestic (*n* = 21). The sample was homogeneous on most of demographic data (Table 1) as no significant differences were observed between amnestic × nonamnestic groups of PD and CO for age, education level, MMSE, MoCA, and BDI. Higher scores of UPDRS, Schwab-England, and LED were documented in nonamnestic PD-MCI group without significance. PD group consisted mainly of men, whereas the controls were predominantly women. The majority of amnestic PD-MCI, nonamnestic PD-MCI, and amnestic CO-MCI did not have vascular risk factor (Table 1).

Neuropsychological data with statistical significance are shown in Table 1. Both PD groups (amnestic and nonamnestic) showed worse performance on several tests when compared to CO. Memory (RAVLT and FMT), attention (RAVLT- trail A1, FMT-incidental memory, and TMT), and executive function (TMT and RAVLT-retroactive interference) impairment were more severe in amnestic PD-MCI group. Nonamnestic PD-MCI presented more severe impairment of executive function (TMT and RAVLT-retroactive interference) when compared with nonamnestic CO-MCI. No differences were observed in language and visuospatial function between the 4 groups.

The data of visual scale score according to MCI subgroup did not allow any logical correlation between PD × CO groups or amnestic × nonamnestic subgroups (Table 1).  Table 2 shows the variables with statistical correlation between MRI visual scales, clinical data, and neuropsychology tests. The following inverse correlations with *p* < 0.05 were observed in the different groups: (a)* amnestic PD-MCI*: (1) GCA scale with age, MoCA, memory (learning FMT and long-term memory FMT), and executive function (learning FMT), (2) MTA scale with UPDRS, and (3) Fazekas scale with memory (learning FMT) and executive function (learning FMT and T1 Stroop); (b)* nonamnestic PD-MCI*: (1) GCA scale with MMSE, executive function (T1 and T2 Stroop), and semantic memory/language (verbal semantic fluency); (c)* amnestic CO-MCI*: (1) GCA scale with executive function and attention (error 3 Stroop), (2) MTA scale with age and executive function (T2 Stroop), and (3) Fazekas scale with executive function (T1 Stroop); (d)* nonamnestic CO-MCI*: (1) GCA scale with education level, MoCA, and memory (recognition memory RAVLT).

## 4. Discussion

Our objective was to evaluate if clinical variables and MRI findings could have some peculiarities in amnestic and nonamnestic MCI in PD comparing with CO group.

An overall assessment of the frequency (descriptive statistic) of MRI visual scales changes according to each MCI subtype (Table 1) did not reveal consistent results. The analysis of each test score relative to the visual scales (statistical inference) provided several correlations (Table 2). Statistically significant inverse correlation was observed between GCA scale and the following neuropsychology scores: MMSE, MoCA, verbal semantic fluency (semantic memory/language), Stroop test (executive function and attention), FMT (memory and executive function), TMT-B (executive function), and RAVLT (memory). The MTA scale correlated with Stroop (executive function) and the Fazekas scale with FMT (memory and executive function) and Stroop test (executive function), according to the subgroup evaluated as described in Table 2.

WMH, evaluated by Fazekas scale, damages cholinergic pathways and results in impairment of cognitive functions. WMH in PD-MCI with attention deficit has already been reported and can be considered a risk factor for MCI in PD patients [15, 16]. The impact of white matter lesions in our study was documented by a correlation between Fazekas scale with memory and executive function in amnestic PD and executive function in amnestic CO.

Cortical gray matter atrophy (CGMA) also has been related to lower neuropsychological scores and as a predictor of dementia in PD-MCI [3]. The CGMA could be related to GCA as cerebral cortex is composed of gray matter. In our sample lower memory score was documented in amnestic PD which presented correlation with GCA; therefore more severe memory impairment could be related to GCA.

The medial temporal lobe is the main structure related to memory, a wide cognitive process [13]. In PD the pathophysiology of memory problems seems to be more complex with impairment retrieval (frontostriatal dysfunction), learning, and recognition deficits [1, 6, 16]. We did not find a correlation between memory impairment and MTA. Probably the MTA scale has a low sensitivity for memory impairment in PD-MCI due to the aforementioned pathophysiology.

We excluded almost 50% of individuals with MCI initially selected due to posterior detection of secondary causes that could justify the cognitive impairment, hence avoiding bias. The use of benzodiazepine and silent stroke in the elderly population is relatively common and these two are the main causes of exclusion in our sample. As a result, there were a relative low number of participants, which can be considered a limitation of the study.

When we prospectively analyzed our data, we might conclude which scales abnormalities in MCI could be a risk factor for developing dementia, and if this is higher in PD group, as such individuals are more prone to develop an unfavorable cognitive outcome.

## 5. Conclusion

Structural abnormalities in MRI were found in PD-MCI and CO-MCI. Evaluation of frequency according to PD-MCI or CO-MCI did not reveal any consistent result. Analyzing each neuropsychological test score with MRI visual scales revealed an inverse correlation with the following cognitive domain: memory, attention, executive function, and language. Visual scales by MRI in MCI should be evaluated by cognitive domain and might be more useful in more severely impaired MCI or dementia patients.

## Figures and Tables

**Table 1:** Sample characteristics and performance in the neuropsychological tests (raw scores).

Variables	PD-MCI (*n* = 44)		CO-MCI (*n* = 44)		*p* ^(a)^
	Amnestic (*n* = 23)	Nonamnestic (*n* = 21)	Amnestic (*n* = 23)	Nonamnestic (*n* = 21)	
	Mean (sd)	Mean (sd)	Mean (sd)	Mean (sd)	
Age (years)	61.61 (6.867)	61.62 (8.047)	61.48 (6.901)	61.24 (6.549)	0.999
Education level (years)	9.61 (4.153)	10.62 (3.556)	11.43 (3.501)	11.24 (4.300)	0.385
Sex% (male/female)	56.5/43.5	71.4/28.6	30.4/69.6	28.6/71.4	—
Vascular risk factor% (yes/no)	40.0/60.0	21.4/78.6	37.5/62.5	54.5/45.5	—
MMSE	27.13 (2.201)	27.81 (1.401)	27.39 (1.158)	27.76 (1.221)	0.559
MoCA	22.0 (3.849)	22.86 (3.410)	21.35 (3.725)	22.67 (3.596)	0.619
BDI	9.52 (6.186)	5.67 (4.386)	8.35 (4.978)	7.10 (4.679)	0.168
Duration of symptoms (months)	76.17 (35.006)	73.14 (38.681)	—	—	0.634^(b)^
L-dopa equivalent dose (mg)	673.09 (295.904)	1284.76 (3072.005)	—	—	0.715^(b)^
UPDRS-III	25.96 (34.458)	67.62 (86.098)	—	—	0.495^(b)^
PDQ39 (total)	31.29 (13.768)	21.89 (13.547)	—	—	0.018^(b)^
Schwab-England scale	82.17 (7.359)	87.14 (5.606)	—	—	0.014^(b)^
MI^(c; 1)^	4.65 (1.152)	5.67 (1.653)	5.26 (1.054)	5.81 (1.250)	0.025
M1^(c; 2)^	7.13 (1.140)	8.14 (1.108)	8.09 (0.996)	9.00 (0.949)	<0.001
M2^(c; 3)^	8.35 (1.071)	9.05 (1.024)	8.43 (1.376)	9.57 (0.598)	<0.001
M5^(c; 4)^	6.74 (1.888)	8.10 (1.640)	8.26 (1.137)	8.95 (0.973)	<0.001
A1^(d; 5)^	4.04 (0.976)	4.62 (1.322)	4.57 (1.647)	5.76 (1868)	0.010
B1^(d; 6)^	3.83 (1.466)	5.19 (1.601)	4.65 (1.824)	5.33 (2.129)	0.015
A6^(d; 7)^	3.96 (2.440)	7.71 (2.704)	6.57 (3.273)	8.71 (2.704)	<0.001
A7^(d; 8)^	3.87 (2.181)	8.10 (2.948)	6.52 (3.475)	10.0 (2.588)	<0.001
LOT^(d; 9)^	10.57 (5.558)	18.90 (7.516)	17.48 (7.609)	17.90 (5.957)	<0.001
RI^(d; 10)^	0.50 (0.261)	0.71 (0.172)	0.70 (0.298)	0.75 (0.167)	0.008
Recognition^(d; 11)^	4.13 (3.912)	10.71 (2.883)	5.09 (4.709)	11.14 (2.833)	<0.001
TMT A^(e; 12)^	68.75 (20.914)	66.71 (19.587)	55.82 (18.213)	49.64 (21.135)	0.003
TMT B^(e; 13)^	231.12 (128.926)	186.83 (79.737)	168.16 (99.767)	147.59 (66.065)	0.028
GCA% ((0) = none/(1) = mild/(2) = moderate/(3) = severe)	30.4/52.2/17.4/0	38.1/57.1/4.8/0	26.1/60.9/13/0	33.3/57.1/9.5/0	—
MTA% ((0) = none/(1) = ↑ of choroid fissure/(2) = ↑ of temporal horn/(3) = ↓ hippocampus/(4) = ↓↓ hippocampus)	52.2/39.1/8.7/0/0	57.1/42.9/0/0/0	56.5/30.4/8.7/4.3/0	57.1/42.9/0/0/0	—
FAZEKAS% ((0) = none or single punctate lesion/(1) = multiple punctate lesions /(2) = confluency lesions/(3) = large confluent)	39.1/34.8/17.4/8.7	38.1/47.6/9.5/4.8	26.1/52.2/13.0/8.7	9.5/52.4/33.3/4.8	—

^(a)^Kruskal-Wallis test; ^(b)^Mann-Whitney; ^(c)^Figures Memory Test; ^(1)^Incidental memory; ^(2)^Learning; ^(3)^Number of figures recalled after 1-minute visualization; ^(4)^Recall of memorized figures after 5 minutes; ^(d)^Rey Auditory Verbal Learning Test; ^(5)^Retrieval of words presented in a list of 15 substantives; ^(6)^Retrieval of words included in the interference list of 15 new substantives; ^(7)^Number of words memorized from list A without reread; ^(8)^Number of words memorized from list A, without reread, after 20-minute interval; ^(9)^Leaning curve of the words during attempts A1 to A5; ^(10)^Retroactive Interference (A6/A5); ^(11)^Correct answers in recognition list; ^(e)^Trail making test; ^(12)^trail A (time in seconds); ^(13)^trail B (time in seconds).

**Table 2:** Spearman correlation.

	PD-MCI amnestic *n* = 23 (*p* value)	PD-MCI nonamnestic *n* = 21 (*p* value)	CO-MCI amnestic *n* = 23 (*p* value)	CO-MCI nonamnestic *n* = 21 (*p* value)
GCA × age	0.467 (0.025)			
GCA × education Level				−0.460 (0.036)
GCA × MMSE^(1)^		−0.516 (0.017)		
GCA × learning FMT^(2)^	−0.440 (0.035)			
GCA × long-term memory FMT^(2)^	−0.485 (0.019)			
GCA × verbal semantic fluency test		−0.454 (0.38)		
GCA × recognition memory RAVLT^(3)^				−0.549 (0.010)
GCA × T1 Stroop^(4)^		0.442 (0.045)		
GCA × T2 Stroop^(5)^		0.614 (0.003)		
GCA × error 3 Stroop^(6)^			0.450 (0.036)	
GCA × TMT-B^(7)^	0.439 (0.036)			
GCA × MoCA^(8)^	−0.521 (0.011)			−0.440 (0.046)
MTA × Age			0.482 (0.020)	
MTA × T2 Stroop^(5)^			0.499 (0.015)	
MTA × UPDRS-III	0.446 (0.033)			
MTA × UPDRS-total	0.454 (0.030)			
FAZEKAS scale × learning FMT^(2)^	−0.446 (0.033)			
FAZEKAS scale × T1 Stroop^(4)^			0.532 (0.009)	

^(1)^Mini-Mental Status Examination; ^(2)^Figures Memory Test; ^(3)^Rey Auditory Verbal Learning Test; ^(4)^naming time (seconds) card 1, Stroop test; ^(5)^naming time (seconds) card 2, Stroop test; ^(6)^number of errors card 3, Stroop test; ^(7)^trail B (seconds), trail making test; ^(8)^Montreal Cognitive Assessment.
